# Supplementary material for: Sources of stress and coping mechanisms: Experiences of maternal health care providers in Western Kenya
Source: PLOS Glob Public Health. 2023 Feb 10;3(2):e0001341. doi: 10.1371/journal.pgph.0001341 (PMC10022275; doi:10.1371/journal.pgph.0001341)
Supplement: Attachment — Submitted filename: Response to PGH- Sources of Stress 11.9.22- final.docx [file pgph.0001341.s001.docx]

**Point-by-point response letter**

We would like to thank the reviewers and editorial staff for the prompt review of our manuscript. Below is the point-by-point response to the reviewers’ and academic editor’s comments. We have also tracked the changes in the manuscript and included a clean version of the paper in the resubmission. We believe these changes have resulted in a stronger and clearer manuscript.

**Journal Requirements**

1. ***Competing*** ***interests***: We have noted that the authors have no competing interests to declare on page 43, line 750-751.
2. ***Conforming to established reporting guidelines***: We have revised our manuscript to better adhere to the MMARS and COREQ. Further, we added a reflexivity statement on page 5, lines 99-106. We note the request from the editorial team to reference a methodological orientation used to underpin the study. We drew on the qualitative philosophy and values of reflexive thematic analysis as described by Braun and Clarke, integrated with codebook thematic analysis, where we used a codebook of deductive and inductive codes. This adaptation was important to enable our inclusive team based approach to coding and analysis. We have described our approach in more detail, including our positionality, the data coding framework development, group approach to analysis, and the use of the socio ecological framework for organizing our findings at the individual, interpersonal, community, and organizational/health systems levels. We did this as we felt these would allow us to identify potential intervention points at each of those levels. Further, part of our approach to the analysis is grounded in our reflexivity- the inclusion of those who collected the data, those who represent the communities in which the study is being conducted- are both integral to the analysis and interpretation process. While we felt no one method fully encapsulates our approach, we heavily drew thematic analysis approach and utilized a group-based analysis and interpretation to build rigor into our analysis.
3. ***Clinical officer*** is the term used to refer to non-physician clinicians who can provide some of the clinical services usually provided by doctors in Kenya (similar to nurse practitioners in the US). We have clarified this in the manuscript.

**Response to peer reviewers**

| **Reviewer 1** | | |
| --- | --- | --- |
| **Reviewers’ comments** | **Response from authors** | **Edited pages in clean manuscript** |
| In the introduction section (line 72), I suggest using a consistent term as in the title “maternal health providers” instead of “maternal health staff”. | We have made this change. | Page 3, line 72 |
| In the settings section, there is a need of more details about the number of health facilities and population of the study. | We initially tried to avoid duplication with previous publications by only summarizing and including citations for where we previously described the study setting. We have, however, included more details about the setting as requested. | Page 5, lines 109-119 |
| In the article that the authors cited, there is information about the number of nurses, clinical officers, and doctors (respectively 32, 19, and 4 100,000 people in the county), however, readers should be able to know the expected number of each professional category (population of the study). This will allow to estimate the response rate and the distribution of responses by profession. | Because of the constant movement of providers in the County, we did not have an exact count of the number of providers in each category, per facility. We were, however, aware that only the referral hospital was likely to have a medical doctor; most other facilities have at least one clinical officer, with two or more nurses or midwives and one or more support staff, based on the facility size. Thus, this informed our recruitment strategy. As noted, all who were invited agreed to participate.  We have provided additional detail on the sampling and response rate. | Page 6 lines 125-144 |
| In addition, it is unclear if some categories of maternal health providers like anesthetists were not available in the county of study. | Only the county referral facility, which has capacity for c-section has an anesthetist. They were however not contacted to participate in the study as they were not available during the interviews. |  |
| Furthermore, there is no information about the total number of health facilities in the county of interest which is essential in determining the generalizability of the results. The authors should provide this important information. | We have now provided more information about the number of health facilities in the county in the study setting. We intentionally selected the high birth volume facilities because of the lower burden of supervising births in the smaller facilities. | Page 5, lines 113-122 |
| Line 109: which participants were from private versus public health facilities? | The distribution of participants from private vs public health facilities is presented in table 1. 14 out of the 101 survey participants, and 4 of the interview participants were from private facilities and the rest public facilities. We have not included type of facility in representative quotes, because of the small number of providers from private facilities, which could potentially identify informants. | Page 9-10 |
| Line 111: How many research assistants? How were they trained? | We have specified number and of research assistants (2) and their training and experience. | Page 6, lines 131-133 |
| Considering the total number of maternal health providers per each professional category (nurses, doctors, clinical officers), how many questionnaires were sent out? How many questionnaires were returned (this will allow to report the response rate in the results section below)? | We did not send out surveys to a recipient list. All surveys were conducted face to face in person. We consulted with the administrative unit leads to identify eligible participants. Research assistants then approached all eligible providers in the facility on the day of the interview and invited them to participate. All agreed to participate giving a response rate of 100%. This has been clarified in the methods. | Page 6, lines 124-144 |
| Line 125: Add a sentence about how the quantitative questionnaire was validated in the current settings. Was any pilot (of the questionnaire) done? Which language was used for the quantitative survey? | We have added a sentence noting that the quantitative study questionnaire was piloted with five (n=5) providers in the county prior to the actual survey. We have also added that our questionnaire was a multi domain survey which included measures previously validated in similar settings.  We have clarified that the survey was administered in English, Swahili, or Luo, based on the participant’s preference. | Page 7, lines 149 - 156 |
| In the data analysis section, a reviewer with expertise in qualitative methods may comment on the choice of the deductive method in comparison to a combined inductive and deductive method especially that there is an existing literature on this topic (I am not an expert in qualitative method, I am not the best person to review this part). | We note this comment and have further elaborated our qualitative analysis approach and orientation in response to other reviewers’ comments below. Of note however, we used a combined deductive and inductive approach, not only a deductive approach. | Pages 7- 8 |
| In the Ethical Approval section, the authors should add the ethical approval numbers from the 2 research ethics boards. | We have added this information. | Page 9, lines 197 - 199 |
| In the results section, the authors should provide the information on the response rate. | We have added this to the methods section as noted above. | Page 6, lines 129-138, 148-149 |
| In the discussion section, the implication about the authors comments on the first limitation is needed after the following sentence “Nevertheless, data were gathered across many facilities representing government, private and other health centers, and among varied informants including a range of cadre responsible for maternal health care provision and supportive roles.” | We have added an additional sentence on the implications of the additional comment on the first limitations. | Page 33, lines 728 - 729 |
| The authors may consider changing the section of limitation to “strengths and limitations” and start with the following sentence about the study’s strength “line 680: The strength of our study is that our analysis and interpretation involved the full study team including those who collected the data, strengthening the rigor of the study and validity of data interpretation.” Then continue with limitations. | We have adjusted the section to reflect this suggestion. | Page 32 - 33, lines 724 - 737. |
| The first sentence of the conclusions “The perennial stressors within health care systems that we and others have identified, calls for the integration of programs to reduce the sources of stress and promote positive coping mechanisms.” should be re-written to reflect the main findings. The information about the authors “we and others” doesn’t need to be written in the first person. | We have made the edits to reflect the study findings and removed references to the team in the first-person. | Page 33, lines 740 |
| **Reviewer 2** | | |
| **Reviewers’ comments** | **Response from authors** | **Edited pages in manuscript** |
| 1.The manuscript could suggest incorporating occupational health’s relevance to the extent of its legal implication. | We have made the edits to reflect this. | Page 32, lines 719 - 721 |
| 2. The discussion could have included experience of well developed nations in combating workplace stress and Coping Mechanisms. | We have added several sentences and literature to address this. | Page 32, 706 - 710 |
| 3. Line 482 Non – work related stress as a cause of mistreatment of patients were mentioned by females’ responders only. It would be interesting to find out what male respondents’ response was. | We note this observation. We have included a quote from a male participant in the manuscript. The qualitative cohort was majority female (67%) and thus the findings reflect the gender balance of the overall sample. We note that we didn’t conduct structured counts of data by sex. Further, the most illustrative quotes were provided, given the limited space in the manuscript. | Page 23, 568 - 572 |
| 4. LINE 360 The multidimensional cause of undesired effect on mother and/or newborn is extremely misunderstood that the providers fear as a wrong perception of incompetence. | We concur with the reviewer’s comment that the quote illustrates an important point that providers fear the consequence of negative outcomes and being perceived as incompetent. We also highlight sections of the discussion that cite and emphasize this point. | Page 29, lines 637- 639 |
| 5.Line 563 Policy level stressors could be well elaborated. | We note the various policy level stressors discussed including lack of adequate incentives and rewards, high turnover, and lack supplies and resources discussed in detail. We have also added in a few sentences regarding the policy level challenge. | Page 27, 604 - 609 |
| 6. If hidden reporting mechanism of occupational stress and its possible causes existed as well as, reporting of colleagues with burnout to the extent of endangering patients, could be put as a suggestion. | We have added this suggestion to the discussion- specifically, to include system levers or reporting mechanisms to prevent negative patient outcomes and safeguard both provider and patient safety. | Page 31, lines 667 – 670 |
| 7. Random / frequent assessment of occupational stress and burn out could be suggested. | We have also included a suggestion for routine or periodic assessments of stress and burnout among providers. |  |
| **Reviewer 3** | | |
| 1.It is not clear what " maternity providers" means - it appears to imply health/clinical workers i.e., doctors, nurse’s lab techs, pharmacists etc. Is this the case? If so, why not call them health workers? Are there other providers of care e.g., CHVs, social workers, patients' relatives etc. More elaboration is need on this. | We acknowledge this comment. We felt the use of maternity health providers best describes both the area of expertise and breadth of skills of the cohort of the study. The study included both clinical (doctors, clinical officers, nurses, midwives) as well as non-clinical support staff (nurses’ aides, cleaners) as a system of providers engaged in the provision of care in the maternal health units/wards of the sites engaged for the study. Other healthcare workers who did not provide maternal health care in these facilities are not included in the study. | |
| 2. Are emotional and psychological as indicated in the manuscript deemed to be separate phenomena? | We acknowledge the similarities in these terms. We define emotional as those narratives having to do with feelings where the impact includes being fearful, scared, worried, or unhappy, whereas psychological is referring to the reported state of informant’s minds- for example, discussions of depression or other ideations which may signal more than just emotions. Since this is a qualitative study and our scope of investigation didn’t extend to establish whether providers were experiencing psychological ailments, we felt including both would be inclusive of both instances of fatigue. | |
| 3. The three 'coping mechanism' themes, how were they arrived at? How are they distinct? | As described in our methods, we conducted an inductive and deductive coding where we began with deductive codes based on the research questions. The parent code “coping mechanisms” was deductively developed as a broad parent code to capture any instances/discussions of how informants cope with or manage stress. Through iterative coding and review of data, we found three distinct approaches to coping: avoidance/not acknowledging stress, managing stress by engaging with others (outside of self), and managing stress by engaging in activities individually. We also informed these inductive codes based on the reported loci of control (as per the participant’s narratives) with some resorting to external coping strategies, while others resorted to individual strategies. | Pages 7- 8 |
| 4. What new information does this study give that is not already more or less documented and quite well known? The authors would benefit from showing the unique information that they are bringing to this subject matter | Although the topic has been previously examined, our study is among the few that qualitatively, using rich narratives, discuss experiences of stress and burnout among maternal health providers in SSA, and in Kenya. We also note (please refer to lines 85-89), that qualitative studies that highlight the pathways and experiences of stress and burnout, as well as coping strategies are critical to identifying intervention points. Given these are likely context specific, studies in multiple settings are needed. We have made edits to reflect this. | Page 4, lines 85-89 |
| 5. Small grammatical errors that need correcting | We have conducted a close copy edit of the manuscript and corrected grammatical and spelling errors. | All pages |
